# Update on Congenital Myopathies in Adulthood

**DOI:** 10.3390/ijms21103694

**Published:** 2020-05-24

**Authors:** George Konstantinos Papadimas, Sophia Xirou, Evangelia Kararizou, Constantinos Papadopoulos

**Affiliations:** 1st Department of Neurology, Eginition Hospital, Medical School, National and Kapodistrian University of Athens, 157 80 Athens, Greece; sopxir@hotmail.com (S.X.); ekarariz@med.uoa.gr (E.K.); constantinospapadopoulos@yahoo.com (C.P.)

**Keywords:** adult onset congenital myopathies, late onset myopathies, inherited myopathies

## Abstract

Congenital myopathies (CMs) constitute a group of heterogenous rare inherited muscle diseases with different incidences. They are traditionally grouped based on characteristic histopathological findings revealed on muscle biopsy. In recent decades, the ever-increasing application of modern genetic technologies has not just improved our understanding of their pathophysiology, but also expanded their phenotypic spectrum and contributed to a more genetically based approach for their classification. Later onset forms of CMs are increasingly recognised. They are often considered milder with slower progression, variable clinical presentations and different modes of inheritance. We reviewed the key features and genetic basis of late onset CMs with a special emphasis on those forms that may first manifest in adulthood.

## 1. Introduction

Congenital myopathies (CMs) constitute a heterogenous group of rare inherited muscle diseases, usually presenting since birth or early in infancy with hypotonia, muscle weakness and skeletal deformities [1,2,3,4]. The first cases of a congenital myopathy were reported in 1956 by Shy and Magee [5], who described five affected members of a family at consecutive generations. Muscle biopsy of all afflicted members showed a characteristic histochemical abnormality resulting from aberrant fibrillary bundles in the center of muscle fibers, which gave the disease the name of “central core” [6]. A few years later, in 1958, D. Reye, a famous Australian pathologist, known for his description of Reye syndrome, observed fractured myofibrils forming irregular rod-like fragments on the muscle biopsy of a 3-y old boy suffering from a myopathy. This finding was initially considered just a processing artefact caused by myofibrils’ retraction and tearing-up of sub-sarcolemmal nuclei and it took some decades to recognize the importance of Reye’s observation [7]. The disorder, known as nemaline myopathy, owes its name to rods (nemaline bodies) and it is of note that the term “nemaline” comes from the Greek word nēma (νῆμα), meaning thread.

The above short historical overview is indicative of the powerful role of muscle biopsy in the diagnosis and nomenclature of CMs at those early years and can absolutely explain their initial histopathological-oriented classification, which, though still in use, tends to be replaced by genetic terms, in the golden era of modern genetics [3,4,8].

The main forms of CMs are the following:

- Nemaline myopathies;
- Core myopathies;
- Centronuclear myopathies;
- Congenital fiber type disproportion (CFTD);
- Myosin storage myopathies.

Although the term “congenital” implies that these diseases present with symptoms at birth, it has become clear, especially under the light of modern genetic technologies, that there are also late-onset forms of CMs, which may be considered in the differential diagnosis of an adult myopathic patient. This is why many experts recommend alternately grouping them with other neuromuscular disorders [3].

Lastly, there are also some conditions, though not falling into the category of inherited myopathies, which may be associated with histological hallmarks of CMs. As an example, nemaline rods may be observed in the sporadic late-onset nemaline myopathy (SLONM), an acquired disorder with subacute proximal muscle weakness, respiratory involvement and dysphagia that may be associated with monoclonal gammopathy of undetermined significance (MGUS) or HIV infection. Apart from the presence of rods, there is no other histopathological similarity between SLONM and congenital nemaline myopathies. However, SLONM should always be considered in the appropriate clinical and pathological setting, as it is one potentially treatable condition [9,10].

So, the aim of the present review is to focus on inherited “paradoxical” late-onset forms of CMs, addressing the dilemma of their possible re-classification and alerting clinicians, who follow adult myopathic patients, to include them in the differential diagnosis.

## 2. Classical Classification

### 2.1. Nemaline Myopathies

**Figure 1:**
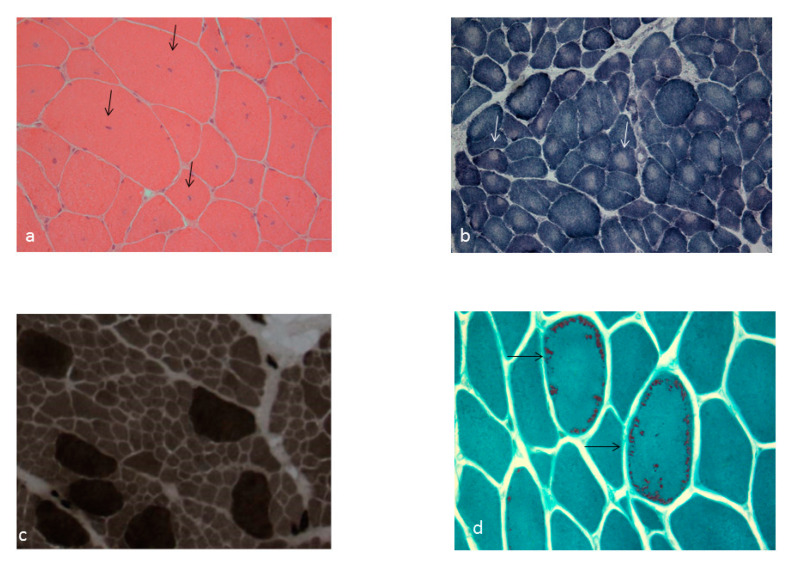
Muscle biopsies showing (**a**) prominent central nuclei (arrow) in a patient with centronuclear myopathy (H&E, ×20), (**b**) prominent central cores (arrow) in a patient with central core disease (NADH, ×20), (**c**) hypotrophy of type 1 muscle fibers (light) relative to type 2 (dark) in a patient with congenital muscle fiber disproportion (ATPase pH 9.4, ×20), (**d**) muscle fibers with nemaline rods (arrow) in a patient with nemaline myopathy (Gomori trichrome, ×40).

The histopathological hallmark of nemaline myopathies is the presence of nemaline rods, visualized in Gomori’s modified trichrome staining (GMT) as dark blue material, which are cytoplasmic inclusions located in the periphery of muscle fibers (Figure 1). They originate at the Z-line of the muscle fiber and mainly contain actin filaments, a-actinin and other Z-disc proteins. It is noteworthy that rods may be better detected on electron microscopy (EM), especially in cases of young patients, where they may be very rare [11]. Rod myopathies are usually inherited as autosomal dominant or recessive traits, but there are also rare sporadic cases. The genes encoding for nebulin (*NEB*) and a-actin (*ACTA1*) are the most common genetic cause of nemaline myopathies, although at least 11 other genes have been related with this disease (*TPM3*, *TPM2*, *MYPN, KLHL40*, *KLHL41*, *LMOD3*, *CFL2*, *TNNT1*, *TNNT3*, *MYO18B*, *KBTBD13*) [12,13]. Most of them are involved in the expression of thin filament-associated proteins of the sarcomere [14]. The majority of nemaline myopathies have their onset at birth and most severe patients may have also respiratory involvement, feeding difficulties and developmental delay. There are, though, some few cases that may be present in childhood or even in adulthood [12,13,14]. Thus, a classification into six subtypes, according to the age of onset and the severity of motor and respiratory symptoms, has been proposed by International Consortium on Nemaline Myopathy (ENMC), with the last three referring to later onset forms [15].

### 2.2. Core Myopathies

Core myopathies encompass the Central Core Disease (CCD) and Multiminicore Disease (MmD), both characterized by the presence of cores, which are areas lacking oxidative activity (Figure 1). CCD is caused by mutations in the ryanodine receptor gene (*RYR1*) and is mostly inherited in an autosomal dominant manner, whereas MmD is mainly caused by mutations in selenoprotein (*SEPN1)* and is usually inherited as an autosomal recessive trait [2,3,4,16]. In CCD, cores are usually single, centrally located or rarely eccentric, and they run the length of the muscle fiber, while in MmD they are smaller and multiple, running along a limited extent of the fiber [17,18]. The phenotypic spectrum of both forms is quite diverse, and especially the susceptibility to malignant hyperthermia in *RYR1*-associated CMs is of important clinical relevance [2,3,4,19,20]. The high incidence of *RYR1* mutations in CMs and the variable, even non neuromuscular, manifestations, called for the need of the 217th ENMC international workshop, which was dedicated to recent advances in the field of *RYR1*-related myopathies [21].

### 2.3. Centronuclear Myopathies

Centronuclear myopathies are a group of hereditary muscle diseases characterized by the presence of abundant centrally placed and/or internalized nuclei, organized in rows in muscle fibers (Figure 1). To date, at least nine different genes (*MTM1*, *DNM2*, *RYR1*, *BIN1*, *MYF6*, *CCDC78*, *TTN*, *SPEG*, *ZAK*), with a variable mode of inheritance (X-linked, autosomal dominant or recessive), have been implicated in centronuclear myopathies [1,2,4]. They can present with variable disease severity and age of onset, ranging from severe congenital myopathy in males, as in the X-linked form of the disease (*MTM1*) to patients with mild, adult onset, myopathy, usually associated with the autosomal dominant forms (i.e., *DNM2*) [1,2]. Most of the genes implicated in centronuclear myopathies are involved in phosphoinositide metabolism, membrane trafficking and remodeling, T-tubule formation, and triad assembly [22,23]. Besides the presence of a central nuclei, additional histopathological findings can point to the underlying gene defect, such as radial arrangement of sarcoplasmic strands (*DNM2)*, necklace fibers in female patients with *MTM1* gene mutations or myofibrillar disorganization in *RYR1* mutations [24].

### 2.4. Congenital Fiber Type Disproportion (CFTD)

The histologic hallmark of congenital muscle fiber disproportion (CFTD) is the hypotrophy of type 1 muscle fibers relative to type 2, in the absence of other structural abnormalities (Figure 1) [1,2]. Brooke and Engel originally described that type 1 fibers should be at least 12% smaller than type 2 for the diagnosis, but now there is consensus that a threshold of at least 35%–40% is more appropriate [25,26]. There is much debate about if CFTD is really a subtype of congenital myopathy, since some patients, if re-biopsied at an older age, will show features such as abundant central nuclei or nemaline rods, allowing them to be reclassified to another diagnosis. Nevertheless, in some cases, type 1 fiber hypotrophy is the predominant, constant and only histologic feature allowing them to be diagnosed with CFTD [26,27]. CFTD is inherited as an autosomal recessive or dominant trait, and genes implicated include *TPM3*, *RYR1*, *ACTA1*, *TPM2*, *SEPN1*, *MYL2*, *HACD1*, *MYH7*, *TTN* and *SCN4A* [4], although the most common causes are mutations in *TPM3* and *RYR1* genes [3]. A family with CFTD and X-linked inheritance, without, at present, an identified responsible gene, has been reported [28]. Patients present with slowly progressive or static generalized weakness, and depending on the underlying gene defect, they may also have respiratory muscle weakness, facial weakness, dysphagia and ophthalmoplegia [3,26].

### 2.5. Myosin Storage Myopathies

The characteristic morphological finding of hereditary myosin storage myopathies is the subsarcolemmal accumulation of hyaline material, exclusively in type 1 fibers, which stain with myosin ATPase, but not with oxidative enzymes [29,30]. Myosins are a large group of proteins, mainly responsible for muscle contraction, although they are also found in other cell types [30]. The genetic basis of myosin storage myopathies are mutations in the rod region of *MYH7* (slow/b-cardiac myosin heavy chain I) gene, which is mostly expressed in heart muscle and slow type I muscle fibers [29,30]. The age of onset is highly variable, ranging from neonatal to adult onset forms, and weakness is usually distal or scapuloperoneal. Respiratory and cardiac involvement are occasionally observed [29,30,31,32,33,34].

## 3. Pathomechanisms of Congenital Myopathies and Genetic Associations

An increasing recognition of the underlying pathophysiological mechanisms of CMs, mainly due to the ongoing application of modern genetic technologies and the considerable clinical and histopathologic overlapping tend to shift the classification to a more genetic basis. Most of the responsible genes for CMs encode for proteins implicated in structure and function of muscle fiber. The basic pathomechanisms and the corresponding implicated genes are the following:

- Thin filament dysfunction, as a result of a primary defect in proteins or modifiers of actin thin filament (*NEB*, *ACTA1*, *TPM2*, *TPM3*, *TNNT1*, *TNNT3*, *LMOD3*, *CFL2*) [21]
- Protein turnover dysregulation, due to defect in Kelch family member proteins, which are important for the proper function of the ubiquitin-proteasome system (*KLHL40*, *KLHL41*, *KBTBD13*) [35]
- Membrane trafficking and/or remodelling defects, caused by mutations in myotubularin (*MTM*) with a central role in membrane identity and protein recruitment, dynamin (DNM2), a key protein for endocytosis [36] and amphiphysin (BIN1) mainly involved in membrane remodeling [37]
- Oxidative stress increase by mutations in selenoprotein (*SEPN1*), which is implicated in modulating EC coupling [38]
- Altered calcium release from sarcoplasmic reticulum stores, due to mutations in skeletal muscle ryanodine receptor 1 (*RYR1*), or rarely ryanodine receptor 3 (*RYR3*) and the dihydropyridine receptor (*CACNA1S*) the voltage-dependent calcium channel serving as a voltage sensor [39,40,41]
- Disruption of cytoskeleton structural integrity caused by mutations in myopalladin (*MYPN*), normally interacting with a-actinin and nebulin [42] or in a-actinin-2 (*ACTN2*), a component of Z-disc with an important role in contractile apparatus integrity [43]
- Aberrant myosin activity, the core element for muscle contraction, due to mutations in myosin heavy chain 7 and 2 (*MYH7*, *MYH2*) [30] and in myosin XVIIIB (*MYO18B*), a newly recognized sarcomere assembly factor for filament alignment [44]
- Destabilization of thick filaments as a result of mutations in titin (*TTN*), which interacts with many sarcomeric proteins and protects sarcomere from overstretching [45]

In summing up, CMs are mostly associated to mutations in genes encoding for proteins involved in skeletal muscle calcium regulation, EC coupling and the myofilament assembly [46]. However, the explosive development of molecular genetics has led to the identification of many new causative genes, suggesting alternative or additional pathomechanisms that may contribute to the etiology of CMs. The main histopathological findings and the causative genes that have been identified for each type to date [4,47] are summarized in Table 1.

## 4. Congenital Myopathies in Adulthood and Classification by Protein and Gene Defect

The diagnosis of CMs in adulthood may be due either to a delay in recognition of symptoms that were present since an earlier stage of development or to a clear adult onset disease. Recent studies have strengthened the assumption that subtle and/or non-specific signs and symptoms (i.e., mild scoliosis, slow runners) may go unnoticed or even attributed to other non-neurological conditions [48]. Listed below by gene and protein defect, are the main characteristics of those forms of CMs that may manifest in adulthood (in Table 1, highlighted in bold are just those CMs subtypes associated with clear adult onset cases).

### 4.1. RYR1-Related Myopathies

Ryanodin 1 is the major calcium release channel from the sarcoplasmic reticulum in skeletal muscle, mediating excitation–contraction coupling and initiating muscle contraction [49,50]. Mutations in the *RYR1* gene have emerged as the leading cause of non-dystrophic myopathies, encompassing a wide spectrum of disease phenotypes, collectively called *RYR*-related myopathies (*RYR*-RM) [51,52] and are the most common cause of late-onset CMs [48]. Histopathological subtypes of *RYR*-RM include CCD [53], centronuclear myopathy [54], CFTD [55], MmD [56], core-rod myopathy [57] and congenital neuromuscular disease with uniform type 1 muscle fibers [58]. Autosomal recessive mutations of the *RYR1* gene are usually associated with early onset weakness and ophthalmoparesis [21,46]. Recessive *RYR1* mutations with adult onset presentations have been rarely reported. These patients may have a history of adverse reaction during an anesthetic procedure and can present with myalgia, asymptomatic hyperCKemia or an adult onset, slowly progressive, proximal lower limb weakness. Muscle biopsy may show marked type 1 muscle fiber predominance, core and multiminicore pathology or “dusty cores” corresponding to irregular areas of myofibrillar disorganization and a reddish-purple granular material deposition, with uneven oxidative stain, devoid of ATPase activity [51,59,60,61,62,63]. There are also a few reports of patients with adult onset episodic paralysis or muscle weakness and a positive McManis test for periodic paralysis [64] and adult onset patients with progressive hand weakness and jaw contractures with core pathology and compound heterozygous or heterozygous mutations in the *RYR1* gene [65]. Autosomal dominant *RYR1* mutations have a variable age of onset, ranging from birth to the 6th decade of life [51] and are associated with a variable phenotypic spectrum including malignant hyperthermia susceptibility, rhabdomyolysis, proximal weakness and possibly mild facial weakness. Ophthalmoparesis, unlike recessive cases, is not common [48,51,60,66,67]. Rare presentations include a late-onset axial myopathy with bent spine syndrome due to the prominent involvement of paraspinal muscles [68,69] and an adult-onset calf predominant, core myopathy [70]. Muscle biopsy findings in late onset dominant cases can reveal an increased number of internalized nuclei, type 1 muscle fiber predominance, central cores, mini/multiminicores or non-specific findings. Serial muscle biopsies can show a progression of histopathological alterations to the final development of central cores [51]. Lower limb muscle MRI in dominant *RYR1* cases, reveal a rather typical pattern, even in mild cases, with sparing of the gracilis, adductor longus, rectus femoris and semitendinosus muscles in the thighs, while the rest of the musculature show diffuse involvement and a prominent soleus involvement in the legs [71]. Dominant mutations in the *RYR1* gene are found in the majority of individuals with malignant hyperthermia susceptibility (MHS), an allelic disorder to CCD. It is characterized by a predisposition to a hypermetabolic response to volatile anesthetics or to depolarizing agents, such as succinylcholine. Muscle rigidity, rhabdomyolysis, hyperthermia, tachycardia, acidosis and hyperkalemia are among the main clinical and laboratory findings. It is not unusual to diagnose siblings with CCD of an otherwise asymptomatic individual with an episode of malignant hyperthermia [51,72,73,74,75]. MHS has been associated with exercise-induced rhabdomyolysis, [76,77,78,79,80] and recently, *RYR1* mutations have emerged as an important cause, of rhabdomyolysis in healthy persons, even without an association with MHS [81]. These patients usually have no evident muscle weakness on clinical examination, and they can even be very athletic, showing muscle hypertrophy. Occasionally, mild proximal lower limb weakness and subtle signs of an underlying neuromuscular disorder, such as mild ptosis, pes cavus or scoliosis or exercise-related cramps and myalgia have been described. There are various triggers for *RYR1*-related rhabdomyolysis, with exercise being the most important, especially if unaccustomed and performed under extreme environmental circumstances, such as infection and fever. Other factors may be medical or illicit drugs and alcohol consumption. When related to exercise and unlike metabolic diseases, rhabdomyolysis occurs at an interval that can be longer than a day. Muscle imaging is usually normal, while muscle biopsy may show variable findings, ranging from normal or non-specific mild myopathic changes to cores, minicores, internalized nuclei and type 1 muscle fiber predominance, more typical to a congenital myopathy. Dantrolene has been proposed as a potentially preventing agent for episodes of rhabdomyolysis [51,81,82,83,84,85,86,87].

### 4.2. SEPN-Related Myopathies

There are at least 25 genes encoding for selenoproteins, which have mostly oxidoreductase activity and are essential for human health. Mutations in the selenoprotein N (*SEPN1*) gene have been identified as the cause of the following four autosomal recessive diseases, which are classified under the title of the *SEPN1*-related myopathies: rigid spine congenital muscular dystrophy, MmD, CFTD and desmin-related myopathy with Mallory body-like inclusions [88,89]. Despite some clinical differences among the various forms, *SEPN1*-related myopathies share many common clinical features. More specifically, they are usually characterized by hypotonia, rigid spine and respiratory involvement, due to diaphragmatic and/or accessory muscle weakness, which may be disproportionate to limb weakness. Muscle MRI may serve as a useful diagnostic tool, revealing a selective involvement of sternocleidomastoid and semimembranosus muscle [90,91]. The course of the disease is usually rather stable and most adult cases of *SEPN1*-related myopathy described in the bibliography had their first symptoms early in life. Especially for the *SEPN1*-related centronuclear and CFTD myopathy, there is no adult onset case reported to date [92,93,94].

### 4.3. Dynamin 2-Related Myopathies

DNM2 is a member of the family of large GTPases, called dynamins. It is a ubiquitously expressed 100 kDA GTPase, mediating membrane fission in various cellular processes, such as endocytosis and membrane division [95]. Autosomal dominant mutations in the *DNM2* gene cause centronuclear myopathy [36] and dominant intermediate and axonal Charcot–Marie-Tooth disease [96,97]. *DNM2*-related centronuclear myopathy is histologically characterized by the presence of a substantial number of central nuclei, type 1 muscle fiber predominance and hypotrophy and the presence of sarcoplasmic strands in oxidative stains, radiating from the central nucleus to the periphery [98]. Since early descriptions, it was evident that *DNM2*-related centronuclear myopathy can present with mild childhood, adolescent and late-onset forms [36], but also with severe forms of congenital myopathy [99]. *DNM2* mutations are the main cause of adult centronuclear myopathies, with disease onset sometimes reaching the 5th decade of age [100,101,102,103,104,105,106]. Clinical signs indicative of *DNM2*-related centronuclear myopathy are distal muscle involvement, ptosis and/or ophthalmoplegia, reduced or absent tendon reflexes, calf or paraspinal muscle hypertrophy, mild cognitive impairment, jaw contractures or trismus, an elongated face and high arched palate. Patients may present with a restrictive pattern of respiratory involvement [103,104,105,106,107,108]. A selective pattern of muscle involvement in muscle imaging has been reported, including lateral pterygoid and temporalis muscles of mastication, cervical and lumbar paraspinal muscles, the deep forearm compartment in the upper limbs and early involvement of the medial gastrocnemius and soleus with sparing of the peroneal group in lower legs and early involvement of the adductor magnus, biceps femoris, semitendinosus and semimembranosus muscles in the thighs [103,109,110].

### 4.4. BIN1-Related Myopathies

Amphiphysin 2 is a ubiquitously expressed protein encoded by the *BIN1* gene in the locus 2q14. It is involved in membrane remodeling and T-tubule organization [111,112]. Autosomal recessive *BIN1* mutations are reported to cause infantile or childhood onset, usually slowly progressive, congenital myopathy [37,112]. Dominant mutations can present as an adult onset myopathy, with an age of onset ranging from 20 years to the 6th decade, showing slowly progressive proximal lower leg weakness, occasionally associated with mild ptosis and ophthalmoparesis, while patients presenting with only myalgia and elevated creatine kinase levels have been reported. Muscle imaging shows involvement of all distal muscles, mostly of posterior compartment and selective proximal muscles affection, particularly involving adductor longus and sartorius [113,114]. Muscle biopsy findings in autosomal dominant *BIN1*-related myopathies are consistent with those found in recessive cases, with the predominance of rounded, hypotrophic type I fibres and prominent central nuclei, usually clustered in the central part of the fibre. In NADH-TR staining there is a clear central zone with a dark border, corresponding to the nucleus [98,113]. A specific phenotype of autosomal recessive *BIN1*-related congenital myopathy has been reported in Spanish Roma patients, harboring the p.Arg234Cys founder mutation either in homozygosity or rarely in compound heterozygosity with the p.Arg145Cys variant. Disease onset ranged from childhood to the sixth decade and patients presented with proximal limb weakness and ophthalmoplegia, and prominent axial weakness, associated with a rigid spine. Interestingly, abundant myotonic discharges and clinical myotonia were observed in a subset of patients. Muscle MRI in these patients revealed a specific pattern with fat infiltration of paravertebral muscles, the posterior compartment of the thighs, soleus, and medial gastrocnemius. [115].

### 4.5. MTM1-Related Myopathies

Myotubularin 1 is a 3′-phosphoinositides phosphatase involved in muscle cell differentiation and, by interacting with desmin, in regulation of mitochondrial morphology [116,117]. Mutations in the *MTM1* gene cause a severe form of X-linked congenital myopathy, with poor prognosis and a high mortality rate, characterized by severe neonatal weakness, hypotonia, respiratory insufficiency and swallowing difficulties [1]. Less severely affected patients with delayed motor milestones and respiratory involvement, who may survive until adulthood [118], or, rarely, adult onset cases have been also described [119,120,121], mainly characterized by proximal limb-girdle weakness, ophthalmoplegia and myopathic face. Although the course of the disease is slowly progressive, ventilatory support may be required, especially under infectious conditions [119,120,122]. Muscle biopsy in late-onset cases may show predominant, rounded hypotrophic type 1 muscle fibers and an increased number of central nuclei, surrounded by a halo lacking enzymatic activity, with occasionally increased NADH and absent ATPase activity [98,119,120,122]. Heterozygous manifesting female carriers are increasingly recognized, with disease severity ranging from forms resembling affected males [123,124,125] to very late onset myopathy [126]. Skewed X-inactivation pattern has been proposed as the cause for these discrepancies in some studies [125,127] but not confirmed in others [128]. Adult onset female carriers usually present with predominately proximal limb-girdle weakness that may be asymmetric, sometimes associated with ptosis and/or ophthalmoparesis. Weakness is slowly progressive, occasionally leading to loss of ambulation. There can be facial asymmetry, in the form of hemifacial hypoplasia, and asymmetric skeletal growth manifesting as one hand or leg being smaller than the other. Some patients, especially those with early disease onset or with more severe muscular weakness can present respiratory insufficiency, or hemidiaphragmatic paresis has been reported [123,124,126,128,129]. The muscle MRI pattern in those female patients is not fully clarified, but shows an asymmetric generalized proximal and lower leg muscle involvement and left–right asymmetries in skeletal size [123,128]. Muscle biopsy may show type 1 muscle fiber predominance and hypotrophy and an increased number of internalized nuclei. A characteristic finding can be the presence of so-called “necklace” muscle fibers, hence fibers with a sub-sarcolemmal, cytoplasmic basophilic ring of aligned myonuclei, evident with H&E, GMT, PAS and oxidative stains, following the outline of the fiber [1,123,130].

### 4.6. Nebulin-Related Myopathies

The major cause of nemaline myopathy are mutations in *NEB* (nebulin) accounting for more than 50% of cases. Nebulin is a very large protein involved in many different critical functions of muscle cells, such as the regulation of actin length and actin–myosin interaction during muscle contraction [131,132]. The absence of hot spot mutations and the lack of a strong genotype–phenotype correlation make diagnosis and prognosis both difficult and challenging [133]. The phenotypic spectrum of the *NEB*-associated nemaline myopathy (NEM2) is wide, ranging from a usually early onset axial and proximal muscle weakness to rare later onset forms, while a distal involvement may be also observed [134]. The first report of a distal recessively inherited nebulin myopathy was provided by Wallgren-Pettersson et.al., who described seven Finnish patients from four different families, who carried two different missense mutations in *NEB* gene in homozygosity. Most of them had their first symptoms in childhood, while two patients noticed a foot drop in their thirties. Neither fiber 1 predominance nor nemaline bodies were revealed on muscle biopsy [135]. There is a more recent report of an adult patient diagnosed at 65 years, whose first symptoms appeared during the 5th decade of life. He had a slowly progressive general muscle weakness with a predominant involvement of anterior tibial compartment and respiratory insufficiency [136]. The distal nebulin myopathy should be differentiated from other CMs with distal affection, such as those related to *MYH7* or *ACTA1* mutations, or other muscle diseases with anterior compartment involvement, such as myofibrillar myopathies [137].

### 4.7. ACTA1-Related Myopathies

The skeletal muscle α-actin is the main contractile protein of thin filaments in sarcomere and has an essential role in muscle contraction [138]. The clinical features of *ACTA1* disease-causing mutations, which are mostly dominantly inherited, can range from the most common severe phenotype with hypotonia, weakness and respiratory involvement to very rare late onset forms. Muscle biopsy is characterized by a wide spectrum of histopathological abnormalities, including actin aggregates, caps, core-like areas, rods, nemaline and zebra bodies or non-specific myopathic changes [139]. The heterogeneous clinical presentation of *ACTA1*-related nemaline myopathy (NEM3) has been initially emphasized in a cohort, which also included the first adult onset case of a male, who complained of exerting weakness at 42 years, followed by a slowly progressive muscle weakness and swallowing difficulties, while muscle biopsy showed myofibrillar disorganization and nemaline bodies. [140]. The case of a 58-year-old woman with *ACTA1* mutation, presenting with a fascioscapuloperoneal phenotype has been recently described [141]. Except for some mild symptoms as a child, she started to have slowly progressive muscle weakness, myalgia and occasionally dysphagia and dyspnea over the last years. She also had a positive family history, with her mother and daughter showing similar mild symptoms. Muscle pathology consisted of nemaline rods and type 1 fiber atrophy [141]. A scapuloperoneal distribution of muscle weakness has been also reported in a large family with a variable phenotypic spectrum and age of onset. While most affected members had their first manifestations in infancy or childhood, few siblings experienced their first symptoms in adulthood with proximal, but more prominent distal muscular weakness with hypothenar atrophy and foot drop, mild scapular winging and facial involvement with a transverse smile. Muscle biopsy lacked the characteristic rods, showing just type 1 fiber atrophy and non-specific myopathic changes [142]. The respiratory related symptoms, such as dyspnea on effort or recurrent infections, should not be overlooked, even in adult patients with an otherwise mild course. Indeed, acute respiratory failure has been described in few late onset patients with *ACTA1* mutations, who had a disproportionately milder muscle weakness [143].

### 4.8. TPM2 and TPM3-Related Myopathies

The tropomyosins are regulatory proteins of the contractile apparatus and the cytoskeleton, playing a crucial role in the interaction of actin with other actin-binding proteins and myosin [144,145,146]. Mutations in *TPM2* and *TPM3* encoding for β-tropomyosin (βTm) and slow α-tropomyosin (αTm-slow) are rare cause of CMs and have been associated with a number of different entities, mainly CFTD, nemaline myopathy and cap myopathy. Although, the onset of the disease is usually at birth or in early childhood, there are also few adult onset cases [147,148,149,150]. A high degree of phenotypic diversity and intrafamilial variability has been observed in *TPM2*- and *TPM3*-related myopathies. In one of the first reports describing three affected siblings with *TPM3*-related cap myopathy, the clinical presentation was variable and the time lapse between initial symptoms and diagnosis was very long, implying that the clinical course was rather mild. The diagnosis was facilitated by muscle biopsy and muscle MRI findings with gluteal, biceps femoris, soleus and tibialis anterior involvement and masseter muscle hypertrophy [151]. The majority of patients exhibit their first symptoms very early in life (congenital/neonatal onset forms) and even some few adults with prominent symptoms at a later stage had subtle signs of earlier involvement. This was the case of a 57-year-old patient with *TPM3*-related nemaline myopathy with his first respiratory symptoms at the age of 40, who had had muscle weakness since early childhood [152], or similarly in a 56-year-old female with *TPM2*-related CFTD, evaluated for dyspnea and muscle weakness, who had delayed motor milestones, a hoarse voice and an easy fatigability since her early life [153]. It is hypothesized that the mechanism of muscle dysfunction differs depending on the specific *TPM3* or *TPM2* mutation, which may result in either impaired actin binding or abnormal myofilament calcium sensitivity. An illustrative example is the increase in the calcium sensitivity during force generation in patients with the p.K7del *TPM2* mutation, which may explain the absence of muscle weakness in their childhood, while it can also provide a basis for understanding why some causative mutations for CMs may primarily influence contractile function through different mechanisms (cellular stress, disruption in energy metabolism, etc.) with fixed weakness at a later stage [154]. Moreover, the effect of some CM-causing mutations on the contractile properties of sarcomeric proteins can result in muscle hypercontactility, leading to early contractures without weakness [147,148,154].

### 4.9. MYPN-Related Myopathies

A recently recognized genetic cause of nemaline myopathies is due to mutations in *MYPN* gene encoding for myopalladin, a multifunctional protein found at the cardiac and skeletal muscle sarcomeric Z- and I-bands and in the nucleus [155]. The recessively inherited *MYPN*-associated nemaline myopathy is histologically characterized by the presence of intranuclear rods, which may be also observed in ACTA1 mutations and in patients with SLONM [9,156,157]. However, in a recent paper by Lornage et.al, the main structural defect in muscle fibers was the presence of caps, which are well demarcated and peripherally located areas filled with thin filaments and Z-line material. All three patients of that study were adults, although their very first symptoms appeared at a neonatal period or early in childhood, and they run a slowly progressive course thereafter [158]. Overall, few patients have been described to date, usually with a mild childhood to adult onset myopathy, who run a relatively benign course [156,159]. The “hanging big toe” sign due to severe extensor hallucis longus weakness, which is considered an almost pathognomonic finding in MYH7 mutations, has been observed in two siblings, who also had some cardiac involvement [159].

### 4.10. Kelch-Related Myopathies

Kelch proteins belong to a large family of diverse proteins, sharing the presence of a Kelch-repeat domain and they perform many different functions in skeletal muscle cells, involving the control of cell morphology, proteolysis and gene regulation. Mutations in some Kelch-related genes are responsible for some forms of nemaline myopathy [35]. KLHL40 (kelch-like family member 40), a muscle-specific protein containing a BTB domain and a kelch repeat, is mainly implicated in thin filament stabilization and myogenesis [160,161,162]. In a large cohort of patients, *KLHL40* autosomal recessive mutations (NEM8) were identified as a frequent cause of severe nemaline myopathy, even with prenatal symptoms and unfavourable outcome [163]. More recently, *KLHL41* (kelch like family member 40) mutations, encoding for nebulin-interacting homonymous protein, which is important in myofibril maturation, are also associated with mainly severe early onset forms of nemaline myopathy [164,165].

Mutations in *KBTBD13*, another sarcomeric actin-binding protein, belonging to the Kelch-repeat superfamily, are associated with the autosomal dominant nemaline myopathy (NEM6) by altering thin filament structure and disrupting muscle-relaxation kinetics [166,167]. There are only a few cases described to date, with most patients noticing their first symptoms since early childhood. The course of the disease was usually very slowly progressive, and the main symptoms were proximal and rarely distal muscle weakness with a characteristic slowness of movements. Muscle biopsy may reveal rods and myofibrillar disruption resulting in core-like areas, which are different from the sharply punched out areas seen in CCD, while the hypotrophy of type 2 fibers may be considered as the histopathologic hallmark of NEM6, contrary to the type 1 hypotrophy characterizing all the other forms of CMs [168,169]. There is, however, a case report of an adult patient, who started to have his first symptoms since the age of 50, with proximal muscle weakness of upper and lower limbs and diffuse muscle hypertrophy. An unusual involvement of internal regions in thigh muscles, with a “central shadow” sign in rectus femoris and a “zebra” pattern in gluteus maximus, were observed on muscle imaging, while muscle biopsy showed nemaline rods, cores and type 2 fibre hypotrophy [170].

### 4.11. LMOD3-Related Myopathies

LMOD3, a member of leiomodin protein family, is crucial for the thin filament organization in sarcomeres of skeletal muscle cells. A severe subtype of nemaline myopathy, assigned as NEM10, was firstly described in 2014 [171]. There are no reports of adult onset cases to date, while some patients at the milder end of the spectrum, who may reach adulthood, have symptoms such as facial and limb weakness, scoliosis and nasal speech [172]. In a recent paper by Marguet F et al. [173], fingerprint bodies were revealed on electron microscopy, as the main finding on muscle biopsy of a mildly affected adult patient with recessively inherited *LMOD3*-related nemaline myopathy. Fingerprint bodies are usually ovoid-shaped non-membrane packed lamellae concentrically arranged structures. They are non-specific and can be observed in many different pathologic conditions [174,175,176,177].

### 4.12. ACTN2-Related Myopathies

Alpha-actinin-2, a major Z-disk component, plays a pivotal role in maintaining the integrity of the contractile apparatus as well as in various signaling pathways [178]. Heterozygous mutations in the *ACTN2* gene have been reported in rare patients with early-onset progressive myopathy, diffuse muscle atrophy and respiratory involvement with characteristic arrangement of contiguous and subsarcolemmal cores in muscle biopsy. This form of congenital myopathy was designated as “Multiple structured Core Disease” [43]. Interestingly, heterozygous mutations in the *ACTN2* gene have also been described in adult-onset distal asymmetric myopathy with the early involvement of the tibialis anterior muscle and slow progression to proximal muscles. Muscle biopsy in these cases revealed myopathic features, rimmed vacuoles, eosinophilic inclusion bodies and areas with myofibrillar disorganization along with numerous lobulated muscle fibers [179].

### 4.13. TnT-Related Myopathies

Troponin T (TnT), a protein of troponin complex, is involved in sarcomeric contraction depending on calcium level fluctuations, in both skeletal and cardiac muscle. Mutations in the two muscle-specific isoforms, slow skeletal troponin T1 (TNNT1) and fast skeletal troponin T3 (TNNT3), have been associated with rare forms of nemaline myopathy. *TNNT1*- and *TNNT3*-related nemaline myopathies are inherited as an autosomal recessive and dominant trait, respectively and they are characterized by an early onset severe phenotype [180,181]. There is just one report of a novel missense autosomal dominant *TNNT1* mutation in many members of an extended family with a mild phenotype and a remarkable clinical variability. Interestingly, two affected males had their first symptoms at 15 and 20 years of age, respectively and they run a very slow course, with minor walking difficulty or intermittent dysphagia at an old age [182].

### 4.14. CACNA1S-Related Myopathies

The dihydropyridine receptor (DHPR), is a voltage-gated L-type calcium channel, located in the T-tubule. When depolarized, it is activated, inducing the ryanodin receptor-1 opening and the subsequent release of Ca^2+^ from the sarcoplasmic reticulum, leading to muscle contraction [50]. Dominant mutations in the *CACNA1S* gene, that encodes the pore subunit of DHPR, have been linked to malignant hyperthermia susceptibility and hypokalaemic periodic paralysis [183,184], Moreover both dominant and recessive mutations have been linked with classical CM of variable severity, with antenatal to early childhood onset, congenital or early onset hypotonia, delayed motor milestones and progressive muscle weakness. Muscle biopsy may show a characteristic intermyofibrillar network due to SR dilatation, internal nuclei, and myofibrillar disorganization [185,186]. One patient with a dominant mutation in the *CACNA1S* gene and symptom onset at 36 years of age has been reported. This patient presented with rhabdomyolysis, constantly elevated CK levels and core-like structures in muscle biopsy [48].

### 4.15. RYR3-Related Myopathies

Ryanodine receptors (RyRs) mediate calcium release from endoplasmic stores, a necessary step for muscle contraction. Multiple isoforms have been identified to date. Although, RyR1 is the main skeletal muscle isoform and RyR3 the brain isoform, it seems that the latter may be transiently expressed in the earliest phase of muscle development [187]. In a first report by Nilipour Y etal, compound heterozygosity of missense mutations in RYR3 gene were detected in a 22-year-old patient with signs of weakness since early childhood and nemaline rods in muscle biopsy [40]. However, it is still very early to conclude on the exact role of RyR3 in muscle.

### 4.16. Titin-Related Myopathies

The 219th European Neuromuscular Centre (ENMC) in 2016 was devoted to titinopathies, a term used to include a range of different muscle diseases caused by mutations in titin (TTN), the longest known protein in nature, expressed both in skeletal and cardiac muscle. The main function of TTN is to link thick filaments to Z disc and maintain the structural integrity of sarcomeres. Mutations in *TTN* gene with different modes of inheritance have been associated with several cardiomyopathies (dilated, hypertrophic or restrictive) and muscle diseases, ranging from muscular dystrophies (late onset autosomal dominant tibial muscular dystrophy, LGMD R10 or formerly LGMD2J, adult onset recessive proximal muscular dystrophy, childhood-juvenile onset Emery-Dreifuss-like phenotype without cardiomyopathy) to core (MmD with heart disease) or centronuclear-like myopathies (congenital centronuclear myopathy), or other diseases such as the hereditary myopathy with early respiratory failure, the young or early adult onset recessive distal titinopathy and the rare early-onset myopathy with fatal cardiomyopathy [188]. Although some titinopathies are considered exclusively adult diseases, there are some forms, and especially those with histological features of a CM, such as the *TTN*-related centronuclear myopathy and the *TTN*-related MmD with heart disease, with a congenital or early-childhood onset [188]. In fact, it is worth mentioning that, contrary to core or centronuclear myopathies caused by other genes, *TTN*-related CMs may be associated with cardiac involvement, but not ophthalmoplegia and, similarly to other CMs, muscle MRI is a powerful tool in their diagnostic work-up, usually showing selective involvement of posterior thigh, soleus and peroneal muscles [188]. Rarely, patients with *TTN*-related centronuclear myopathy may present at an older age [189]. In a recent report, a 54-year-old female with a recessively inherited *TTN*-centronuclear myopathy, was diagnosed at her 35 with dilated cardiomyopathy and presented muscle weakness many years later. She had some skeletal manifestations (scoliosis and achilles tendon contractures) since childhood, but the full-blown clinical picture developed in adulthood, implying that *TTN*-related cardiomyopathy may manifest at any age and routine monitoring is always crucial for these patients [190]. Overall, titin is a giant protein encoded by a large gene and the interpretation of pathogenicity of genetic variants remains a great challenge. The development of more effective bioinformatic tools is expected to further expand the phenotypic spectrum of titinopathies.

### 4.17. MEGF10-Related Myopathies

Multiple epidermal growth factor-like domains (MEGF10), a protein encoded by the *MEGF10* gene, is expressed in neural tissue, neuromuscular junction and skeletal muscle, where it promotes myoblast proliferation [191,192]. An autosomal recessive *MEGF10*-related CM with minicores was firstly described in severely patients with early onset of the disease [193]. However, in a recent report of two affected sisters, the first sign was a scoliosis in their adolescence, while respiratory insufficiency and muscle weakness appeared in adulthood. Interestingly, one sibling had also myotonic discharges in many muscles on EMG, without clinical myotonia [194]. The phenotype of *MEGF*-related myopathies was further expanded with the description of an adult patient with myofibrillar pathology, but without minicores [195].

### 4.18. ΜYH7-Related Myopathies

Myosin heavy chain 7 (*MYH7*)-related myopathies are caused by mutations in the *MYH7* gene, which encodes the β-cardiac myosin heavy chain protein. This protein is expressed mostly in the cardiac ventricles, but also in slow type I muscle fibers. These diseases are usually inherited as an autosomal dominant trait, although there are also rare recessive or sporadic cases. The causative mutations in the *MYH7* gene can occur in all three domains of the protein (head, neck and rod) and the resulting phenotype, which is wide, including cardiomyopathies, skeletal myopathies and both, is related to the affected region [29,196]. Especially *MYH7*-related skeletal myopathies can be subdivided in two allelic disorders: myosin storage myopathy (OMIM#608358) and Laing early onset distal myopathy (OMIM#160500) [29,196].

Myosin storage myopathy, formerly known as hyaline body myopathy, due to the characteristic hyaline bodies on muscle biopsy, is caused by mutations in the rod domain of *MYH7* [29]. The age of onset greatly varies, ranging from infancy to adulthood, while muscle weakness is usually proximal in upper limbs and distal in lower limbs with foot drop. There may be also a characteristic pseudohypertrophy of the calves, scapular winging and scoliosis [29,31,32,33,197]. There are, however, rare reports of patients with pronounced proximal muscle weakness [31,198] or even no weakness at all. The case of a 46-year old man with late onset proximal weakness and his 26-year old son with talipes cavus and calf pseudohypertrophy [31] or the report of a three generation family, with the index patient experiencing the first symptoms at around 40 years and her offspring in early childhood [32], are indicative of an intrafamilial heterogeneity on both clinical phenotype and age of onset. The course of the disease may be stable or usually slowly progressive. Respiratory insufficiency may occur [30]. Although cardiac involvement is not common [199], reports of adult onset patients with different degrees of cardiomyopathy do exist [200,201]. Muscle biopsy typically shows hyaline bodies, which are pale eosinophilic subsarcolemmal areas in H&E, exclusively found in type 1 muscle fibers. They lack oxidative activity, but are positive in ATPase at pH 4.6 and are immunoreactive to slow/ β cardiac myosin [33]. Muscle MRI usually reveals a characteristic pattern with pronounced involvement of the posterior thigh compartment, with relative sparing of the semitendinosus and fatty infiltration of the medial gastrocnemius, tibialis anterior, extensor hallucis longus and extensor digitorum longus in the legs [33].

Laing (Gowers–Laing) distal myopathy is caused by dominant mutations, usually in the exons 32-36 of the *MYH7* gene [29,202,203]. Despite the term “early onset distal myopathy”, the first symptoms may appear later in life until adulthood [204,205,206], although in most cases, the disease presents in childhood with weakness in ankle dorsiflexion and the characteristic “hanging big toe”. Calf hypertrophy, finger extensor and neck flexor involvement or even facial weakness may be present in the course of the disease [207,208], while cardiac involvement is variable [203,204,206,207,208,209,210]. Muscle pathology shows non-specific and variable findings. The most common is the fiber size variation, nuclei internalization and type 1 fiber atrophy, while other, less frequent alterations may be fiber type 1 or 2 predominance, fiber type disproportion [206,211], cores, minicores and mitochondrial disturbances [206,210,211]. Contrary to myosin storage myopathy there are no protein aggregates, while rimmed or non-rimmed vacuoles with filamentous inclusions have been reported [30]. Muscle imaging may reveal fatty replacement, especially in the tibialis anterior and extensor hallucis longus muscles at the early stages, with a later thigh muscle involvement over the course of the disease [29,204,205].

### 4.19. MYH2-Related Myopathies

Mutations in the *MYH2* gene may be dominant or recessive. The dominant MyHCIIa myopathy, alternatively called “autosomal dominant myopathy with congenital joint contractures, ophthalmoplegia and rimmed vacuoles”, (OMIM#605637) or “Hereditary inclusion body myopathy type 3” (IBM3) starts prenatally with congenital contractures that may reverse in childhood and patients may eventually lose ambulation [30]. Interestingly, adult patients may show total ophthalmoplegia in contrast to children that may just have a restriction in upward gaze [30]. Muscle pathology may reveal minor changes, with a more characteristic involvement of type 2A fibers, which are both fewer and smaller. Myofibrillar alterations can be also observed, while dystrophic changes, rimmed vacuoles and protein aggregates may be encountered especially in adults [29,30]. Recessive mutations in MyHCIIa are associated with early onset mild generalized weakness with ophthalmoplegia, while muscle biopsy reveals myopathic changes and total absence of the type IIa fibers without rimmed vacuoles [212].

### 4.20. HADC1-Related Myopathy

3-hydroxyacyl-CoA dehydratase 1 (HADC1) is an endoplasmic reticulum (ER)-resident enzyme involved in the synthesis of very long chain fatty acids [213]. Mutations in the *HADC1* gene have been reported in a large consanguineous family with congenital myopathy and muscle fiber type disproportion. All members of this family showed severe hypotonia with gradual improvement at birth and the oldest patient, at the age of 35 years, had normal muscle tone and strength, absent tendon reflexes and residual pes cavus [214].

### 4.21. SCN4A-Related Myopathy

Dominant gain of function mutations in the α-subunit of the skeletal muscle voltage-gated sodium channel (Nav1.4) gene (*SCN4A*) are associated with a variety of muscle phenotypes, including potassium-aggravated myotonia, hypokalaemic periodic paralysis, paramyotonia congenita and hyperkalaemic periodic paralysis, while recessive loss of function mutations are linked to congenital myasthenic syndrome and classical CM [215]. Although the majority of patients with *SCN4A*-related CM present either with a severe clinical picture or a more “classical” CM [216,217], milder phenotypes with minimal muscular complaints, including exertional shortness of breath without significant muscle impairment until adulthood, have been reported [218].

### 4.22. Other Genes Implicated in CMs

Many other genes have been rarely associated with CMs, but large cohorts are still lacking and there are no reports of late onset patients associated with them until now (Table 1) [4,219,220,221]. However, it is expected that the application of modern genetic technologies will increase the diagnostic yield and will broaden the phenotypic spectrum of CMs.

## 5. Conclusions

More than 30 different genes have been associated with CMs to date. They are implicated in many different functions, mainly in calcium homeostasis, thin–thick filament assembly, intracellular membrane trafficking and remodeling and oxidative stress [1,2,3,4,222]. From a histopathological point of view, the most prevalent form is nemaline myopathy, but based on genetics, *RYR1* mutations are the most common genetic cause with many different phenotypic features [223]. Although the term “congenital” implies the appearance of symptoms from birth, there are about 13% of childhood onset and 4% of adult onset cases [224]. In general, most patients with congenital myopathy share some common clinical features, such as hypotonia, elongated face, high arched palate, respiratory involvement and normal to mildly elevated CK [1,2,3,4]. A distal phenotype in *NEB*-associated nemaline myopathy and *DNM2*-associated centronuclear myopathy seems to be more common in late-onset CMs [133,225,226], and similarly a scapuloperoneal pattern may be also observed in late onset *ACTA-1*-related nemaline myopathy [142]. Cardiac involvement is generally not a great concern, as CMs are associated with a low risk of heart abnormalities, with the possible exception of some few forms, such as the *MYH7*- and *TTN*-related CMs, where a regular cardiac follow-up is highly recommended [46,227]. According to AHA recommendations, a cardiac assessment should be initially performed at the time of presentation, with a follow-up depending on the presence of any abnormal finding or any suspicious symptom [228].

Overall, late onset CMs still remain a great diagnostic challenge for the clinicians, mainly due to the still-unidentified genetic causes and the phenotypical overlap with other types of myopathies, such as limb-girdle muscular dystrophies, making correct diagnosis more complicated. However, a high suspicion index and new advanced genetic approaches will both allow a better diagnostic accuracy, so that adult neurologists will increasingly have to cope with patients, who had an adult-onset CM or who developed their first symptoms earlier in life and transitioned into adulthood.

## Figures and Tables

**Table 1:** Summary of the genes and proteins implicated in congenital myopathies (CMs) and their main histopathological associations (highlighted in bold are those genes that have been also associated with adult onset CMs).

Causative Genes	Protein	Mode of Inheritance	Main Histopathological Findings
***ACTA1***	Alpha actin	AD, AR	nemaline rods (also intranuclear), cores, CFTD, actin aggregates, caps, zebra bodies
*ACTN2*	Actinin alpha 2	AD	cores, rimmed vacuoles, eosinophilic inclusions, lobulated muscle fibers
***BIN1***	amphiphysin	AD, AR	central nuclei
***CACNA1S***	Calcium channel voltage-dependent, L type, alpha 1S subunit	AR	central nuclei, cores
*CCDC78*	Coiled-coil domain-containing protein 78	AD	cores, central nuclei
*CFL2*	Cofilin 2	AR	nemaline rods
***DNM2***	Dynamin 2	AD	central nuclei, radiating sarcoplasmic strands
*FXR1*	FMR1 autosomal homolog	AR	Cores
*HACD1*	Protein tyrosine phosphatase-like	AR	CFTD
***KBTBD13***	Kelch repeat and BTB (POZ) domain containing 13	AD	nemaline rods, cores, type 2 hypotrophy
*KLHL40*	Kelch-like family member 40	AR	nemaline rods
*KLHL41*	Kelch-like family member 41	AR	nemaline rods
*LMOD3*	Leiomodin 3	AR	nemaline rods, fingerprint bodies
***MEGF10***	Multiple EGF-like-domains 10	AR	minicores
***MTM1***	myotubularin	XR	central nuclei, necklace fibers
*MYF6*	Myogenic factor 6	AD	central nuclei
*MYH2*	Myosin, heavy chain 2	AD, AR	few and small type 2 fibers, rimmed vacuoles
***MYH7***	Myosin, heavy chain 7	AD, AR	cores, CFTD, myosin storage, rimmed vacuoles
*MYL2*	Myosin light chain 2	AR	CFTD
*MYO18B*	Myosin XVIIIB	AR	nemaline rods
***MYPN***	Myopalladin	AR	nemaline rods (also intranuclear)
***NEB***	Nebulin	AR	nemaline rods
***RYR1***	Ryanodine receptor 1	AD, AR	cores (minicores), central nuclei, CFTD
*RYR3*	Ryanodine receptor 3	AR	nemaline rods
*SCN4A*	Sodium channel voltage-gated, type IV, alpha	AR	CFTD
*SEPN1*	Selenoprotein N1	AR	minicores, CFTD
*SPEG*	SPEG complex locus	AR	central nuclei
***TNNT1***	Slow troponin T	AR	nemaline rods
*TNNT3*	Fast troponin 3	AR	nemaline rods
*TPM2*	Tropomyosin 2	AD, AR	nemaline rods, CFTD, caps
*TPM3*	Tropomyosin 3	AD, AR	nemaline rods, CFTD, caps
*TTN*	Titin	AR	cores, central nuclei, CFTD
*ZAK*	mitogen-activated protein triple kinase	AR	central nuclei

AD: autosomal dominant, AR: autosomal recessive; XR: X-linked recessive, CFTD: congenital fiber type disproportion.
